# Oxytocin biases men but not women to restore social connections with individuals who socially exclude them

**DOI:** 10.1038/srep40589

**Published:** 2017-01-12

**Authors:** Xiaolei Xu, Shuxia Yao, Lei Xu, Yayuan Geng, Weihua Zhao, Xiaole Ma, Juan Kou, Ruixue Luo, Keith M. Kendrick

**Affiliations:** 1 Key Laboratory for Neuroinformation, Center for Information in Medicine, School of Life Science and Technology, University of Electronic Science and Technology of China, Chengdu 610054, PR China

## Abstract

We normally react to individuals who exclude us socially by either avoiding them or increasing our attempts to interact with them. The neuropeptide oxytocin can promote social bonds and reduce social conflict and we therefore investigated whether it facilitates more positive social responses towards individuals who exclude or include us. In a double-blind, placebo-controlled, between-subject design 77 healthy Chinese male and female participants received intranasal oxytocin (40 IU) or placebo before playing a modified virtual ball-tossing game with three fictitious partners who either showed exclusion, inclusion or neutral behavioral interactions with them. Results showed that both male and female subjects threw the ball more often to individuals who excluded rather than included them, although oxytocin did not alter this or awareness/feelings of exclusion or inclusion. However, when subjects returned a week later males, but not females, in the oxytocin group exhibited an increased liking for, and preference for playing again with, players who had previously excluded them. This oxytocin effect was positively associated with independent traits. Our findings suggest that in a collectivist culture oxytocin may promote the desire of males, but not females, with a stronger independent orientation to rebuild social connections with individuals who have previously excluded them.

Being excluded or rejected is an aversive and painful social experience even when observing others suffering from it^1^,^2^. The pain caused by rejection has been shown to share common brain networks including the neural alarm system and somatosensory areas which respond to physical pain^3^,^4^,^5^. Previous studies have shown that social rejection threatens individuals’ fundamental needs, such as belonging, self-esteem, control and having a meaningful existence^6^. The negative effect of social rejection even impacts cognitive function, for example performance in a saccade task^7^, and increased ability to detect response conflicts^8^.

The aversive experience imposed by social rejection results in immediate and long-term threats to feelings of belonging^9^,^10^. Thus, rejected individuals tend to rebuild social bonds with others to satisfy their needs to belong^11^, become more sensitive to cues signaling potential social exclusion, such as fake smiles, and avoid being excluded in other social interaction situations^12^.

Previous studies have demonstrated that the hypothalamic neuropeptide oxytocin (OXT) plays a critical role in promoting social bonds and prosocial behaviors^13^,^14^ and can also reduce social conflict^15^. Importantly, OXT can have anxiolytic effects in terms of reducing stress and anxiety in human and other animal species^16^,^17^,^18^,^19^ and may have therapeutic potential for anxiety disorders^20^,^21^. However, while OXT can act as an anxiolytic and appears to reduce amygdala responses to fear-evoking stimuli^22^,^23^,^24^, consistent with it facilitating either tolerance or avoidance of negative emotional situations, it can in some circumstances enhance reactivity towards and memory for them and therefore potentially trigger greater approach and conciliatory behavior^25^,^26^. Thus, OXT might potentially play a role either in facilitating greater tolerance or avoidance by subjects of individuals who reject them socially, or alternatively facilitate greater approach towards them in an attempt to rebuild positive social connections. This might particularly be influenced by cultural differences with members of Asian collectivist cultures being more likely to respond positively towards excluders rather than rejecting them and reacting less strongly to social exclusion compared to more independently oriented cultures^27^. Indeed, whereas the rs53576 OXT receptor polymorphism is associated with emotional support seeking in Americans, it is not in Koreans^28^. This same polymorphism is also associated with differential responses to social stress^29^ and social exclusion^30^.

A number of previous studies have investigated associations between OXT and social rejection using the Cyberball game paradigm. In this paradigm subjects play a ball tossing game with players via the internet who either exclude or include them during the game and, unbeknownst to them, are actually computer controlled^31^. Two studies have shown that plasma OXT concentrations rise significantly in healthy controls, but not in Borderline Personality disorder or depression patients, after the experience of being excluded in this game^32^,^33^ and another found an association between the rs53576 (G-carriers) polymorphism of the OXT receptor gene and emotional responses to exclusion in the game^30^. One study has reported that intranasal OXT promoted prosocial behavior towards a victim of social rejection in individuals who experienced low levels of maternal love withdrawal^34^. Other studies have shown that OXT treatment made subjects from an independent culture, but with a more collectivistic orientation, retain a higher sense of social comfort when facing ostracism^35^, and increased social affiliation and cooperation in individuals with social anxiety disorder and low attachment avoidance^36^. Thus OXT effects in relation to social rejection may be modified by inter-individual differences in social traits and attachment experience and security. However, another study found no direct effect of OXT on social rejection in the context of the Cyberball game, although it did increase the desire of individuals to play the game again with players who had included as opposed to rejected them^37^. Thus the overall impact of OXT on responses to individuals who socially exclude or include us remains unclear and may be strongly influenced by personal experience and characteristics and potentially also cultural differences.

Previous studies investigating OXT effects on social exclusion or inclusion of the subjects themselves using the Cyberball paradigm have either investigated inclusion and exclusion effects separately in different rounds of the game^35^,^37^, or have included an additional modulatory factor by giving subjects financial incentives for performance^36^,^38^. All previous studies have also only focused on OXT effects during the game, or immediately after it, rather than including a longer term follow up of potential effects after concentrations of the peptide are no longer elevated. In the current study we therefore included three playing partners in the game who either excluded or included or were neutral towards subjects in terms of the frequency of their ball throws to them. In this way we could evaluate the possible differential impact of OXT on responses to individuals who socially excluded or included subjects during the same game. Subjects were also not incentivized by additional monetary payment for performance. As with other studies the effects of playing the game on subjects’ mood and other social parameters were assessed using the positive and negative affective schedule (PANAS) and fundamental needs questionnaire (FNQ). Additionally, to investigate the potential impact of OXT treatment during the experience of playing the game on subsequent behavior subjects returned a week after the initial game and received a surprise memory test for the players they had encountered as well as rating their liking for them and preference for playing with them again. In view of reports by other studies of OXT effects being influenced by gender^39^,^40^,^41^,^42^,^43^ we included both male and female subjects. We also investigated possible modulatory effects of collectivism on OXT effects suggested by a previous study carried out on subjects from an independent orientation culture^35^. Individual differences in collectivism and independence orientations were measured using the independence and collectivism scale (ICS). We hypothesized that if OXT primarily acts to reduce social conflict, and is influenced by a collectivism orientation, it should motivate subjects to focus more on excluders to rebuild social connections and less on includers with whom they already have such connections. Thus OXT should particularly enhance participants’ memory for and liking of excluders and increase their desire to play with them again. If on the other hand OXT is acting to promote greater tolerance or avoidance of players who exclude them, and therefore focus more on those who included them, then it should make includers more memorable, likeable and preferable to play again with than excluders.

## Results

### Questionnaires

Independent t-tests on scores showed that there were no significant differences between participants in the two treatment groups for any of the different behavior trait, social experience and mood questionnaires completed immediately prior to the experiment (see Table 1). There were also no significant differences in these questionnaire scores between male and female participants (see Supplementary Table S1).

### Behavior during the Cyberball task

**Figure 1:**
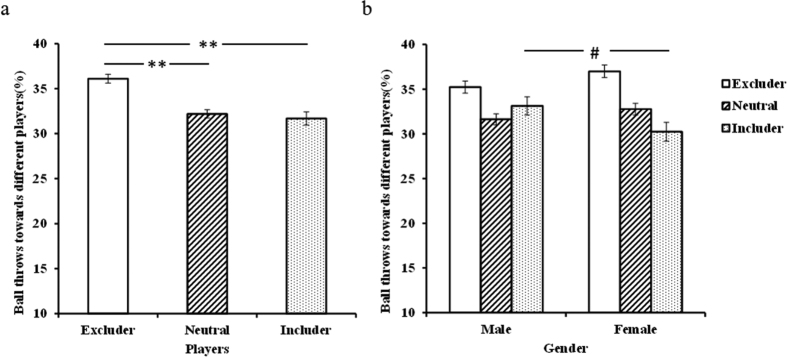
Histograms show mean ± SEM percentage of ball throws made by the subjects in the combined PLC and OXT groups to players who either excluded or included them or were neutral for (**a**) male and female subjects combined and (**b**) separately. (**p < 0.01, *p < 0.05, ^#^*p* = 0.052).

After calculating the number of balls that subjects threw to the other players respectively, a 2 (treatment: PLC/OXT) × 3 (players: excluder/includer/neutral) × 2 (gender: male/female) repeated ANOVA was performed. This analysis revealed a significant main effect of players (*F*_2,146_ = 11.95, *p* < 0.001, 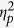 = 0.141) and a players × gender interaction (*F*_2,146_ = 3.26, *p* = 0.041, 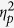 = 0.043). *Post-hoc* Bonferroni-corrected paired comparisons showed that participants threw more balls to players who excluded than included them (excluders: 36.11 ± 0.49% (mean ± SE), includers: 31.69 ± 0.73%, *p* < 0.001, Cohen’s *d* = 0.947) or were neutral (neutral: 32.20 ± 0.45%, *p* = 0.001, Cohen’s *d* = 0.787, Fig. 1a). Further analysis of the interaction indicated that male participants threw more balls to the includer players than females did (Males: 33.14 ± 1.02%, Females: 30.24 ± 1.06%, *p* = 0.052, Cohen’s *d* = 0.457 – Fig. 1b).

### Immediate post-task behavioral assessments

The PANAS scores before and after treatments and performing the Cyberball task were analyzed using paired t-tests but showed no significant differences (see Table 1) between the groups, indicating that OXT treatment did not differentially influence the mood of participants. However, there was a significant decrease in both positive (*t* = 5.42, *p* < 0.01, Cohen’s *d* = 0.748) and negative PANAS scores (*t* = 14.28, *p* < 0.01, Cohen’s *d* = 2.167) across groups in the second test. This may indicate that both groups were experiencing a more neutral mood after the task had been completed. There were also no overall group differences in participants’ FNQ scores in relation to their experience of playing the Cyberball game, other than participants in the OXT group reporting significantly stronger feelings of being involved in the game (*t* = −2.27, *p* = 0.026 see Table 1). Overall subjects in both treatment groups had low FNQ scores for negative emotional responses to the game and high ones relating to positive ones, indicating that in general subjects found playing the game a positive emotional experience despite the exclusion component.

A 2 (treatment: PLC/OXT) × 2 (gender: male/female) Chi-square test (χ^2^) was carried out on participants awareness of either being excluded or included by other players (Q1 and Q3, see Methods). This revealed no significant treatment or gender differences in awareness of inclusion or exclusion (*p* > 0.5 in all cases), although a greater proportion of subjects reported being aware of being included than excluded (see Table 1). A 2 (treatment: OXT/PLC) × 2 (rating: accepted/excluded feeling) × 2 (gender: male/female) repeated ANOVA on strength of feeling scores (Q2 and Q4, see Methods) revealed a main effect of rating (*F*_1,73_ = 169.92, *p* < 0.001, 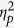 = 0.699) with participants reporting stronger feelings of being included than excluded by others (see Table 1: included rating: 6.36 ± 0.19, excluded rating: 2.50 ± 0.19, *p* < 0.001, Cohen’s *d* = 2.353). There were no other significant main effects or interactions.

### Memory and preference behavior assessments after 1 week

Subjects from both treatment groups had a relatively low mean recognition accuracy of the faces of players from the games played a week before (60–70%). There was no significant difference in recognition accuracy for familiar (previous Cyberball players) and novel faces in the PLC vs OXT groups in the surprise memory task (*t* = 1.86, *p* = 0.067, Cohen’s *d* = 0.430). A 2 (treatment: OXT/PLC) × 3 (recognition of players face pictures: excluder/includer/neutral) × 2 (gender: male/female) repeated ANOVA analysis did not reveal any main or interaction effects. Thus recognition accuracy was similar across player types and was not influenced by OXT.

**Figure 2:**
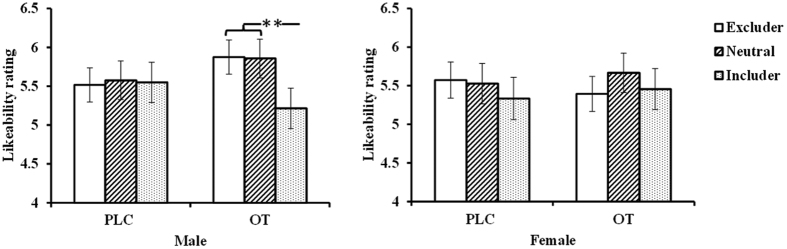
Histograms show mean ± SEM likeability ratings for subjects in OXT and PLC groups for face pictures of excluders, include and neutral players 1 week after the original Cyberball game (**p < 0.01).

A 2 (treatment: OXT/PLC) × 2 (face pictures: familiar/novel) × 2 (gender: male/female) repeated ANOVA on subjects’ likeability ratings revealed a main effect of face pictures (*F*_1,73_ = 33.85, *p* < 0.001, 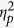 = 0.317) and a likeability × gender interaction (*F*_1,73_ = 4.32, *p* = 0.041, 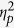 = 0.056). *Post-hoc* Bonferroni-corrected paired comparisons showed that subjects gave higher likeability ratings to faces of the familiar players than to those of novel individuals (familiar: 5.55 ± 0.11, novel: 5.03 ± 0.11, *p* < 0.001, Cohen’s *d* = 0.550). Both genders showed this effect (males: familiar = 5.60 ± 0.16, novel = 4.90 ± 0.15, *p* < 0.001, Cohen’s *d* = 0.773; females: familiar = 5.49 ± 0.16, novel = 5.16 ± 0.16, *p* = 0.011, Cohen’s *d* = 0.331). A 2 (treatment: OXT/PLC) × 3 (face pictures: includer/neutral/excluder) × 2 (gender: male/female) repeated ANOVA found a main effect of face pictures (*F*_2,146_ = 4.43, *p* = 0.014, 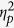 = 0.057) and a treatment × face pictures × gender interaction (*F*_2,146_ = 3.57, *p* = 0.031, 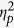 = 0.047). *Post-hoc* Bonferroni-corrected paired comparisons revealed that participants rated likeability of the face pictures of familiar neutral players significantly more than those of includers (neutral = 5.66 ± 0.13, includer = 5.39 ± 0.13, *p* = 0.031, Cohen’s *d* = 0.240). In addition, OXT significantly increased likeability ratings given by males, but not females, for the faces of the familiar excluders compared to those of includers (OXT male: excluder = 5.88 ± 0.22, includer = 5.22 ± 0.26, *p* = 0.001, Cohen’s *d* = 0.611; OXT female: excluder = 5.40 ± 0.23, includer = 5.46 ± 0.27, *p* = 0.744, Cohen’s *d* = 0.066) and for neutral players (OXT male: neutral = 5.86 ± 0.25, includer = 5.22 ± 0.26, *p* = 0.002, Cohen’s *d* = 0.567, Fig. 2; OXT female: neutral = 5.67 ± 0.26, includer = 5.46 ± 0.27, *p* = 0.307, Cohen’s *d* = 0.204). Increased likeability ratings for excluders were not significantly correlated with the number of times subjects threw the ball to them in the original game in either treatment group (*p* > 0.817 in all cases).

**Figure 3:**
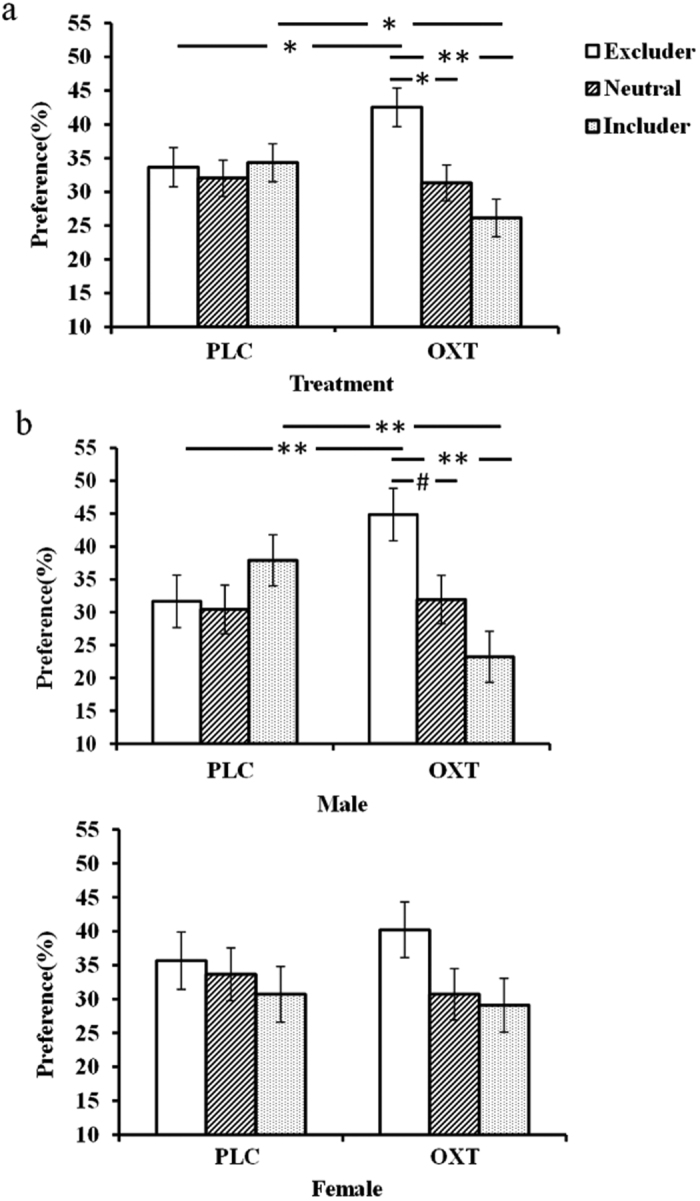
Histograms show mean ± SEM preference choice for subjects in OXT and PLC groups for face pictures of excluders, includers and neutral players 1 week after the original Cyberball game. (**a**) For males and females combined and (**b**) separately. (**p < 0.01, *p < 0.05, ^#^*p* < 0.06).

A 2 (treatment: PLC/OXT) × 3 (player: excluder/includer/neutral) × 2 (gender: male/female) repeated ANOVA was also performed to investigate differential preferences for playing again with specific types of players. Results showed a marginal main effect of player (*F*_2,146_ = 3.01, *p* = 0.052, 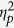 = 0.040), and a significant treatment × player interaction (*F*_2,146_ = 3.14, *p* = 0.046, 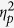 = 0.041). *Post-hoc* comparisons with Bonferroni correction showed that both groups tended to prefer to play again with excluders rather than includers (excluders:38.10 ± 2.04%, includers: 30.23 ± 1.98%, *p* = 0.090, Cohen’s *d* = 0.438) and that OXT significantly increased this preference (PLC: 33.66 ± 2.91%, OXT: 42.53 ± 2.87%, *p* = 0.033, Cohen’s *d* = 0.508) and correspondingly decreased the preference for includers (PLC: 34.31 ± 2.83%, OXT: 26.14 ± 2.79%, *p* = 0.043, Cohen’s *d* = 0.482, Fig. 3a). While there was no significant treatment × player × gender interaction (*F*_2,146_ = 1.43, *p* = 0.243, 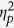 = 0.019), an exploratory analysis revealed that OXT only significantly increased preference for excluders in male but not female participants (Male: PLC: 31.67 ± 3.11%, OXT: 44.86 ± 2.88%, *t* = −3.12, *p* = 0.003 Cohen’s *d* = 1.012; Female: PLC: 35.65 ± 5.50%, OXT: 40.20 ± 4.62%, *t* = −0.64, *p* = 0.528, Cohen’s *d* = 0.216) and decreased their preference for includer players (PLC: 37.92 ± 3.95%, OXT: 23.19 ± 2.74%, *t* = 3.06, *p* = 0.004, Cohen’s *d* = 0.993; Female: PLC: 30.71 ± 4.43%, OXT: 29.09 ± 4.61%, *t* = 0.25, *p* = 0.802, Cohen’s *d* = 0.085 - Fig. 3b). Increased preference ratings for excluders in males were not significantly correlated with the number of times subjects threw the ball to them in the original game in either treatment group (*p* > 0.138 in all cases).

### Association between oxytocin effects and collectivism/independent orientation

**Figure 4:**
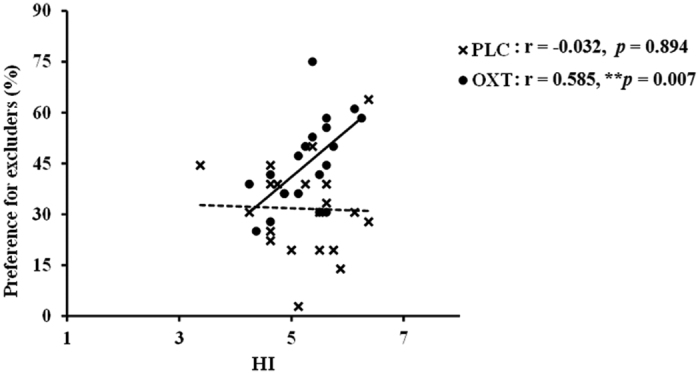
Graphs show correlations between horizontal independence scores on the independence and collectivist scale (ICS) and preference for playing again with excluders for male subjects in the OXT and PLC groups. (**p < 0.01).

We next specifically investigated associations between OXT effects on increased likeability and preference for playing again with excluders, particularly in males, and ICS scores to assess potential modulation by collectivism or independent orientations. As expected subjects in both treatment groups scored higher overall for collectivism compared to independence (see Table 1). For the four sub-scales of the ICS (vertical collectivist- VC, vertical independent- VI, horizontal collectivist- HC and horizontal independent- HI) male subjects under OXT showed a significant positive correlation between preference for playing again with excluders and HI score (r = 0.585, *p* = 0.007), whereas those under PLC did not (r = −0.032, *p* = 0.894, Fisher’s z = 1.987, *p* = 0.047 – see Fig. 4). This association with HI was not seen in female subjects (PLC – r = 0.057, *p* = 0.829; OXT – r = 0.053, *p* = 0.83). Thus males, but not females, in the current study show a greater effect of OXT in promoting an increased preference for re-engaging with excluders if they also show stronger independent traits. Interestingly males, but not females, from both treatment groups showed a positive correlation between their overall collectivism score and the proportion of balls they threw back to the excluders in the original game (males: PLC group r = 0.456, *p* = 0.043; OXT group r = 0.454, *p* = 0.044; females: PLC group r = −0.008, *p* = 0.976; OXT group r = −0.097, *p* = 0.692). This was primarily driven by the horizontal component scores in males (HC-HI: PLC r = 0.529, *p* = 0.017; OXT r = 0.413, *p* = 0.07).

## Discussion

Overall our results showed that OXT did not either influence subjects’ choice of throwing the ball in the Cyberball game to players who either excluded or included them, or their awareness of or feelings towards being included or excluded. Interestingly, in both OXT and PLC groups, subjects threw a significantly greater proportion of balls to individuals who excluded them in the game. However, one week after the game playing experience subjects originally treated with OXT exhibited an increased liking of individuals who had previously excluded them and also a greater preference for playing with them again. This OXT effect was only significant in males and was strongest in subjects scoring higher in independent traits. Thus overall our results suggest that when males play simultaneously with those who both include and exclude them, OXT treatment makes them subsequently more likely to try and re-establish connections with those who have previously excluded rather than included them, particularly if they have a higher independent orientation.

In agreement with previous studies on subjects from independent cultures OXT did not influence patterns of play with either includers or excluders during the Cyberball game in Chinese subjects^35^,^36^,^37^. There was also no evidence from PANAS and FNQ measures taken immediately after the game for any effect of OXT on influencing either subject’s negative or positive emotional reactions or their feelings of self-esteem, sense of belonging, control or meaningful existence. However, under OXT subjects did report increased feelings of involvement in the game. Overall subjects in both groups reported greater positive than negative emotional reactions to the game in their FNQ scores and this was also reflected by them being more aware of being included than excluded during it, and giving higher ratings for their feelings of inclusion rather than exclusion. This pattern of relative insensitivity to social exclusion is similar to that reported by other studies on collectivist cultures^27^. However, our modified version of the Cyberball game had both excluder and include players in each round and this may also have contributed to reduced feelings of exclusion in our subjects. It would be necessary to compare subjects from both collectivist and independent cultures in the same study to establish whether a genuine cultural difference exists. Interestingly though, despite apparently being relatively unaware of being excluded during the game, subjects in both treatment groups actually threw the ball significantly more to excluder players, indicating by their actions that they perceived their exclusion during the game, albeit at a relatively unconscious level.

In Chinese collectivistic culture there is an emphasis on the importance of group harmony so that individuals show greater moderation in distributing any kind of resource to avoid conflict and embarrassment and to promote group harmony and solidarity^44^. On the other hand individuals from independent cultures tend to look after themselves and to ignore group interests if they conflict with personal desires^45^. This cultural difference may explain why both male and female Chinese subjects in the current study interacted more often with excluders during the game. A study on subjects from an independent culture using a similar modified Cyberball paradigm reported that they progressively threw more balls to includer rather than excluder players, which suggests that there may be cultural differences in responses^38^. However, unlike our current study where different players were involved in each round of the game, this latter study always had the same players. Furthermore the Andari *et al*. study^38^ paid subjects money for every ball they received and this may have increased the motivation for them to throw more to individuals who were more likely to throw back to them.

While there was no gender difference in terms of the greater proportion of balls thrown to excluding players during the Cyberball game, in males this was positively correlated with a collectivist orientation whereas in females it was not. Thus it is possible that Chinese male and female subjects had different motivations for playing more with excluder players during the game, with males perhaps doing so in order to try to bring outsiders into the social collective and reduce their potential threat, whereas in females it may perhaps have derived from a greater personal sense of worth as a result of an altruistic action.

Our findings that OXT treatment increased a subsequent preference to play again with excluder players is in contrast to a previous study on subjects from an independent culture which reported that intranasal OXT increased the preference for subjects to play again with includers rather than excluders immediately after the game^37^. However, this latter study involved a paradigm where subjects played separate games with includers and excluders, and it is also likely that OXT concentrations were still elevated at this time following initial treatment. Thus while this might also indicate a cultural difference in OXT effects we can’t rule out the possibility that Chinese subjects would also have shown a preference for playing again with includers if we had tested this immediately after the game, when OXT concentrations were still elevated, rather than 1 week later. However, the possibility of a genuine culture difference would need to be established in a study incorporating subjects from both collectivist and independent cultures.

There is considerable research showing that we are more sensitive to negative emotional events and that they are preferentially processed by the brain relative to neutral and positive ones^46^,^47^. Our findings that male subjects in the OXT group exhibited a significantly increased liking for and willingness to play again with individuals who had previously excluded them might therefore be due to an effect of the peptide in enhancing the emotional salience of these individuals during the game. However, there was no indication of improved subsequent overt recognition accuracy for excluders, and recognition accuracy for players of all types in the game was only slightly above chance (60–70%). There was also no evidence for the increased preference or likeability of excluders being associated with the number of times subjects actually threw the ball to them during the game itself, so the preference is unlikely to be due to greater initial interactions during the game. Thus the effect of OXT treatment during the game may have been to produce an unconscious positive bias towards excluder players which resulted in subjects exhibiting an increased preference and likeability for them even one week later, and even though they showed only a relatively weak capacity for overt recognition of the players themselves. Possibly OXT is in some way strengthening a relatively automatic perception-action link in males that sub-serves the social function of identifying individuals who we are at risk of being rejected or threatened by and responding positively towards them in order to restore social contact and avoid conflict. On the other hand, we cannot completely rule out the possibility that the emotional salience of the excluders was in some way increased by OXT given some previous evidence that it can enhance implicit but not explicit memory for faces^48^.

We did not have an a priori hypothesis that there would be gender differences in the current study although the sex-specific effects of OXT we found provide further support for the growing number of studies suggesting that it may play rather different functional roles in males and females^39^,^40^,^41^,^42^,^43^. While the general pattern of behavior during and immediately after the Cyberball game was broadly similar in male and female subjects the significant increases in preference and likeability for excluding players a week after the original game only occurred in males. This may reflect a strategy to reduce the risk of further exclusion since a previous study has shown that individuals are more likely to engage in social reconnection efforts, such as exhibiting more reciprocity and more trust to their partners, if they feel they are at risk of exclusion^49^. However, while effect sizes observed in significant OXT-evoked changes in males were medium to large in size, there was obviously a reduction in statistical power necessitated by including gender as a factor and thus sex-dependent effects may need further confirmation in a larger study.

Thus OXT may particularly promote attempts to restore social connections following social rejection in males rather than females to help reduce the risk of further social exclusion and potential conflict. This is in broad agreement with previous studies showing that OXT promotes positive social behavior in men, but not women, following couple conflict^40^; only increases activity in striatal brain reward regions in men in response to reciprocated social cooperation^41^; only decreases amygdala and insula activity in response to unreciprocated social cooperation^39^ and decreases risk aversion in men^50^. Taken together with our current findings this suggests that OXT may particularly increase the motivation for men, but not women, to exhibit prosocial behavior towards others who are socially uncooperative or represent a conflict threat. Furthermore, the findings from previous studies were from subjects in independent orientation cultures and in our current study OXT effects were strongest in individuals from a collectivist culture with higher independence orientation scores. Thus OXT may particularly facilitate attempts to restore social connections and reduce the risk of social conflict in men who have a greater independent orientation, perhaps because such individuals are normally less motivated to do this compared to those with a stronger collectivist orientation. Given increasing evidence for the influence of specific OXT-receptor polymorphisms on responses to social exclusion and stress, as well as potential cultural differences in this respect^28^,^29^,^30^,^51^, future studies are needed to elucidate their modulatory roles.

There are several limitations in the current study. First, we did not ask participants to rate their likeability of and preference for replaying with other players immediately after the Cyberball game. Given that OXT increased the likeability and preference choice for excluders even after one week, it is possible that this effect might also have occurred immediately after the Cyberball game. Second, the modification of the Cyberball game integrated both inclusion and exclusion during each round and this may have reduced the degree of exclusion experienced by the participant’s due to potential compensatory effects of simultaneously being included. This could have contributed to the observed effects and future studies would in particular need to consider whether OXT has similar effects in Chinese subjects following a stronger experience of exclusion during the game.

## Methods

### Participants

Seventy-seven healthy students (40 males, all right-handed) were recruited from the University of Electronic Science and Technology of China and were randomly assigned to receive either PLC (n = 20 males and 18 females, mean ± sem age = 22.5 ± 0.4 years) or OXT (n = 20 males and 19 females; age = 22.1 ± 0.3 years, *t* = 0.73, *p* = 0.468) treatment using a double-blind between-subject design. No subjects reported having any current or previous neurological or psychiatric problems and none were taking any medication for at least 4 weeks before or during the whole study. All of female subjects reported having regular menstrual cycles and were not pregnant (subjects were all offered pregnancy tests if required). None of the female subjects reported taking oral contraceptives. Using cycle length information we calculated that 24 participants were in the luteal phase (PLC – n = 13; OXT – n = 11) and 13 in the follicular phase (PLC – n = 5; OXT – n = 8; PLC *vs*. OXT, *t* = 0.90, *p* = 0.376). All subjects were asked to abstain from caffeine and alcohol consumption during the 24 hours before the experiments. The study was approved by the ethical committee of the University of Electronic Science and Technology of China and the experiments were carried out in accordance with the latest revision of the Declaration of Helsinki. Each participant signed written informed consent and was paid 80 RMB for participating.

### Experimental procedure

When subjects arrived at the laboratory, the experimenter firstly took their head shot photos with a neutral facial expression and they were informed that these photos would be used in subsequent experimental tasks. Next subjects were instructed to complete a series of questionnaires including the Positive and Negative Affect Schedule (PANAS), Beck Depression Inventory (BDI), Self-Esteem Scale (SES), State-Trait Anxiety Inventory (STAI), the Revised Cheek and Buss Shyness Scale (RCBSS), the Social Provision Scale (SPS), the Adult Autism Spectrum Quotient (ASQ), the Individualism and Collectivism Scale (ICS) and the Interpersonal Sensitivity Measure (IPSM). Details of the Chinese versions of these questionnaires and Cronbach’s alpha values for subjects in the current study are given in the supplementary section. Subjects were then randomly assigned to receive either intranasal administration of OXT (40IU – Oxytocin-spray, Sichuan Meike Pharmacy Co., Ltd; 5 puffs of 4IU per nostril with 30 s between each puff) or placebo (PLC - identical sprays with the same ingredients other than OXT) which they self-administered 45 minutes before the Cyberball task using a standard protocol^52^. There is currently some debate concerning whether behavioral and neural effects of intranasal OXT are mediated via direct central or indirect peripheral routes^53^, although many reports in both humans^25^ and monkeys^54^,^55^,^56^,^57^ have consistently found increased concentrations in cerebrospinal fluid and the presence of radiolabelled peptides in the brain in rats after intranasal administration^58^,^59^.

The Cyberball task paradigm was similar to the original version^31^. In our paradigm there were four players, including three virtual players (player 1, 3, and 4) and the participant (always player 2). A total of 18 (9 female) different virtual players were used with 6 (3 female) different players of each type. The pictures of all the virtual players were rated (9-point Likert scale) independently by 19 judges (10 male) and were matched for each type of player in terms of likeability, trustworthiness and arousal. Subjects were told that this task aimed to practice their mental visualization skills and they were instructed to throw the ball to others by clicking on their icons when they received it. The Cyberball game was played for approximately 5 min and consisted of six rounds with a total of 60 throws in each one (i.e. 360 throws overall). During each round subjects played with different players who they believed were randomly assigned by the game website. All subjects were told that the experiment included four different universities and that six students from each university were taking part simultaneously. In this pre-programmed Cyberball game in each round there were always a bad player (excluder) who only threw once to the subject, a good player (includer) who threw to them 70% of the time and a neutral player who threw an equal number of times to the subject and the other two players (i.e. 33.3% of the time to the subject). Immediately following this task subjects were asked four questions: Q1 “Were you aware that some players threw the ball to you less than to others during the game?” (Yes or No); Q2 “When they threw less balls to you, how much did you feel you were being excluded? (9-point Likert scale: 1, not at all; 9, very much)”; Q3 “Were you aware that some players threw the ball more often to you than to others?” (Yes or No); Q4 “When they threw more balls to you, how much did you feel you were being included? (1, not at all; 9, very much)”. Subjects then completed the Fundamental Needs Questionnaire (FNQ)^60^ and the PANAS for a second time to test for whether OXT per se had altered their mood (PANAS) or their feelings about participation in the game (FNQ).

One week after the initial Cyberball experiment subjects returned to complete a further three tasks but with no additional treatment. First, a surprise memory test was administered where 36 face pictures (18 from people who had played before with the subject and 18 new ones, randomly intermixed) were presented on a screen. After each picture subjects had to respond whether they had played with the person shown in the picture last week or not by pressing one of two keys (“Yes” or “No”) and were then asked to rate how much they liked them on a 1 to 9 scale. Next the subjects were given a preference task which consisted of 36 trials where three pictures of previous Cyberball players were presented simultaneously on each trial and they were asked to choose which one they would like to play with again. In each case the familiar players comprised an excluder, includer and neutral one from the first experiment but in different groupings from those used originally. Subjects indicated their choice by pressing one of the three keys representing the three individual players. For all of these tasks subjects had no time constraints for making responses. After they completed the whole experiment, all subjects were asked whether they had any doubts that the other Cyberball players were real, and all confirmed that they thought they were. Subjects were also asked to guess whether they had received OXT or PLC treatment but their responses showed that they were unable to do so better than chance.

### Statistical analysis

In all cases statistical analyses were performed using SPSS 18.0 software (SPSS Inc., Chicago, Illinois, USA). Differences between questionnaire scores in the OXT and PLC groups were analyzed using independent t-test. No corrections were made for multiple tests. Treatment effects were analyzed primarily using 3-factor ANOVAs (individual factor details provided in the Results section) and where significant interactions occurred post-hoc analyses were carried out using a Bonferroni correction in SPSS. Treatment differences in responses to Q1 and Q3 by Chi-square test (χ^2^) and 2-factor ANOVA for ratings on Q2 and Q4. Correlations between ICS scores and behavior in the two treatment groups were performed using the Pearson test and differences in correlations between the groups were analyzed using Fisher’s Z. In an initial exploratory analysis we found no evidence for significant menstrual cycle effects on any measures taken and so this was not included as a factor analyses.

## Additional Information

**How to cite this article**: Xu, X. *et al*. Oxytocin biases men but not women to restore social connections with individuals who socially exclude them. *Sci. Rep.* **7**, 40589; doi: 10.1038/srep40589 (2017).

**Publisher's note:** Springer Nature remains neutral with regard to jurisdictional claims in published maps and institutional affiliations.

## Supplementary Material

Supplementary Information

## Figures and Tables

**Table 1:** Questionnaire and rating scores for participants in PLC and OXT group before treatment and after treatment and performing the Cyberball game (mean ± SEM).

Measurements	Placebo	Oxytocin	*t*-value	*p*-value
**Before treatment**				
Self-Esteem Scale (SES)	30.8 ± 0.7	32.2 ± 0.6	−1.51	0.135
Beck Depression Inventory (BDI)	7.5 ± 0.9	6.8 ± 0.9	0.54	0.594
State-Trait Anxiety Inventory (STAI)-State	38.9 ± 1.5	37.1 ± 1.4	0.92	0.361
State-Trait Anxiety Inventory (STAI)-Trait	41.4 ± 1.2	38.6 ± 1.0	1.77	0.081
Adult Autism Spectrum Quotient (ASQ)	21.2 ± 0.7	19.5 ± 1.0	1.38	0.172
Social Provision Scale (SPS)	48.8 ± 1.0	50.4 ± 1.3	−0.98	0.331
Interpersonal Sensitivity Measure (IPSM)	95.9 ± 1.4	94.9 ± 1.2	0.57	0.568
Cheek and Buss Shyness Scale (RCBSS)	36.7 ± 1.1	34.2 ± 1.5	1.35	0.182
**Positive & Negative Affect Schedule (PANAS)**				
Positive	31.2 ± 1.0	33.1 ± 0.8	−1.65	0.104
Negative	20.1 ± 1.0	21.1 ± 0.8	−0.79	0.431
Individualism and Collectivism Scale (ICS)-HI	2.7 ± 0.1	2.6 ± 0.1	0.76	0.450
Individualism and Collectivism Scale (ICS)-VI	3.2 ± 0.1	3.5 ± 0.1	−1.96	0.053
Individualism and Collectivism Scale (ICS)-HC	2.6 ± 0.1	2.4 ± 0.1	0.94	0.348
Individualism and Collectivism Scale (ICS)-VC	3.1 ± 0.1	3.1 ± 0.1	0.07	0.947
**After treatment and playing Cyberball game**				
Positive & Negative Affect Schedule (PANAS)				
Positive	27.2 ± 1.3	27.3 ± 1.2	−0.02	0.982
Negative	10.7 ± 0.6	10.9 ± 0.5	−0.22	0.829
**Fundamental Needs Questionnaire (FNQ)**				
Belonging	3.96 ± 0.2	3.75 ± 0.2	0.69	0.494
Control	3.84 ± 0.1	3.76 ± 0.2	0.27	0.786
Self-esteem	4.94 ± 0.2	4.96 ± 0.2	−0.08	0.937
Meaningful-existence	4.02 ± 0.2	3.68 ± 0.2	1.34	0.184
Mood-bad	2.95 ± 0.4	2.18 ± 0.4	1.47	0.147
Mood-good	6.76 ± 0.4	6.79 ± 0.3	−0.07	0.946
Mood-sad	2.76 ± 0.4	1.95 ± 0.3	1.65	0.104
Mood-happy	6.87 ± 0.3	6.82 ± 0.3	0.11	0.910
Mood-nervous	3.03 ± 0.4	2.44 ± 0.4	1.14	0.256
Mood-relax	6.97 ± 0.4	7.44 ± 0.4	−0.91	0.366
Mood-exciting	5.71 ± 0.4	5.23 ± 0.4	0.90	0.374
Mood-drowsy	3.84 ± 0.4	3.62 ± 0.4	0.39	0.698
Ancillary-enjoy	6.24 ± 0.3	6.44 ± 0.3	−0.43	0.670
Ancillary-angry	2.82 ± 0.4	2.21 ± 0.4	1.20	0.235
Involved	7.18 ± 0.3	8.00 ± 0.2	−2.27	0.026*
Received ball	4.21 ± 0.2	4.10 ± 0.2	0.34	0.735
Q1- awareness of exclusion (%)	34.21 ± 0.1	28.21 ± 0.1	0.56	0.575
Q2- feelings of exclusion (1–9)	2.74 ± 0.3	2.26 ± 0.3	1.30	0.199
Q3- awareness of inclusion (%)	60.53 ± 0.1	64.10 ± 0.1	−0.32	0.750
Q4 – feelings of inclusion (1–9)	6.08 ± 0.3	6.64 ± 0.2	−1.5	0.137
