# Supplementary material for: Oxytocin biases men but not women to restore social connections with individuals who socially exclude them
Source: Sci Rep. 2017 Jan 12;7:40589. doi: 10.1038/srep40589 (PMC5227992; doi:10.1038/srep40589)
Supplement: Supplementary Information [file srep40589-s1.pdf]

Oxytocin biases men but not women to restore social connections with individuals who socially exclude them

Xiaolei Xu, Shuxia Yao, Lei Xu, Yayuan Geng, Weihua Zhao, Xiaole Ma, Juan Kou, Ruixue Luo, &Keith M. Kendrick*

Key Laboratory for Neuroinformation, Center for Information in Medicine, School of Life Science and Technology, University of Electronic Science and Technology of China, Chengdu 610054, PR China

* Address for correspondence: Keith M Kendrick, No. 4, Section 2, North Jianshe Road, Chengdu 610054, China. Tel.: +86-28-83201358. Fax: +86-28-83201358. Email: k.kendrick.uestc@gmail.com

The authors declare no competing financial interests.

English versions of questionnaires used in this study are the Positive and Negative Affect Schedule (PANAS)[1], Beck Depression Inventory (BDI)[2], Self-Esteem Scale (SES)[3], State-Trait Anxiety Inventory (STAI)[4], the Revised Cheek and Buss Shyness Scale (RCBSS)[5], the Social Provision Scale (SPS)[6], the Adult Autism Spectrum Quotient (ASQ)[7], the Individualism and Collectivism Scale (ICS)[8] and the Interpersonal Sensitivity Measure (IPSM)[9].

Chinese versions of questionnaires used and Cronbach's alpha values

Existing Chinese language versions of the following scales were used: Positive and Negative Affect Schedule (PANAS)[10], Beck Depression Inventory (BDI)[11], Self-Esteem Scale (SES)[12], State-Trait Anxiety Inventory (STAI)[13], the Revised Cheek and Buss Shyness Scale (RCBSS)[14], the Adult Autism Spectrum Quotient (ASQ)[15], the Individualism and Collectivism scale (ICS)[16].

We carried out a standard translation and back-translation to provide Chinese versions of the Social Provision Scale (SPS) (Cronbach's alpha = 0.891) and The Social Interpersonal Sensitivity Measure (IPSM) (Cronbach's alpha = 0.782).

Cronbach's alpha for the questionnaires in our sample (n=77):

SES: 0.850; BDI: 0.825; STAI-TOTAL: 0.935 - STAI-State: 0.920 -STAI-Trait: 0.844; ASQ: 0.740; SPS: 0.891; IPSM: 0.782; RCBSS: 0.890; PANAS-TOTAL: 0.833 - PANAS-Positive: 0.746 - PANAS-Negative: 0.696; ICS-TOTAL: 0.746 - ICS-HI: 0.728 - ICS-VI: 0.715; ICS-HC: 0.843- ICS-VC: 0.626.

The values are all in close agreement with the previous larger scale studies cited above.

References

1. Watson, D., Clark, L. A. & Tellegen, A. Development and validation of brief measures of positive and negative affect: the PANAS scales. *J. Pers. Soc. Psychol.* **54,** 1063-1070 (1988).
2. Beck, A. T., Steer, R. A. & Carbin, M. G. Psychometric properties of the Beck Depression Inventory: Twenty-five years of evaluation. *Clin. Psychol. Rev.* **8,** 77-100 (1988).
3. Robins, R. W., Hendin, H. M. & Trzesniewski, K. H. Measuring global self-esteem: Construct validation of a single-item measure and the Rosenberg Self-Esteem Scale. *Pers. Soc. Psychol. Bull.* **27,** 151-161 (2001).
4. Barnes, L. L. B., Harp, D. & Jung, W. S. Reliability generalization of scores on the Spielberger state-trait anxiety inventory. *Edu. Psychol. Meas.* **62,** 603-618 (2002).
5. Hopko, D. R., Stowell, J., Jones, W. H., Armento, M. E. & Cheek, J. M. Psychometric properties of the revised Cheek and Buss shyness scale. *J. Pers. Assess.* **84,** 185-192 (2005).
6. Cutrona, C. E. & Russell, D. W. The provisions of social relationships and adaptation to stress. *Adv. Pers. Relationsh.* **1,** 37-67 (1987).
7. Baron-Cohen, S., Wheelwright, S., Skinner, R., Martin, J. & Clubley, E. The autism-spectrum quotient (AQ): Evidence from asperger syndrome/high-functioning autism, malesand females, scientists and mathematicians. *J. Autism. Dev. Disord.* **31,** 5-17 (2001).
8. Singelis, T. M., Triandis, H. C., Bhawuk, D. P. & Gelfand, M. J. Horizontal and vertical dimensions of individualism and collectivism: A theoretical and measurement refinement. *Cross-cult. Res.* **29,** 240-275 (1995).
9. Boyce, P. & Parker, G. Development of a scale to measure interpersonal sensitivity. *Australas. Psychiatry.* **23,** 341-351 (1989).

10. Lin Qiu, Xue Zheng & Yanfei Wang. Revison of the positive affect and negative affect scale. *Chinese Journal of Applied Psychology*. **14,** 249-254 (2008).

11. Yuxin Zhang & Yan Wang. The reliability and validity of Beck Depression Inventory. *Chinese Mental Health Journal*. **4,** 164-168 (1990).

12. Ping Wang, Hua Gao, Jiayu Xu, Jinju Huang & Chengjiang Wang. The reliability and validity of Self-esteem Scale. *Shandong Archives of Psychiatry.* **11,** 31-32 (1988).

13. Wenli Li & Mingyi Qian. Revised version of State-Trait Anxiety Inventory in Chinese college students. *Acta Scientiarum Naturalium, Universitatis Pekinensis*. **31,** 108-114 (1995).

14. Xiangdong Wang, Xilin Wang & Hong Ma. Rating scales for mental health (Revised edition, in Chinese). *Beijing: Chinese Mental Health Journal Publisher*. 375-378 (1999).

15. Mengrong Liu. Screening adults for Asperger syndrome and high-functioning autism by using the autism-spectrum quotient (AQ)(Mandarin version). *Bulletin of Special Education*. **33,** 73-92 (2008).

16. Renzhi Huang, Shuqiao Yao, Abela, J. R., Leibovitch F. & Mingfan Liu. Key dimensions and validity of the Chinese version of the Individualism – Collectivism Scale. *Chinese Studies*. **2,** 1-7 (2013).

Table S1. Ages and questionnaire scores for male and female participants in the combined PLC and OXT groups (mean ±SEM)

Measurements	Male	Female	*t*-value	*p*-value
Age	22.6 ±0.4	21.9 ±0.3	1.36	1.171
Self-Esteem Scale (SES)	31.4 ±0.7	31.6 ±0.7	-0.31	0.759
Beck Depression Inventory (BDI)	6.5 ±0.9	7.9 ±0.8	-1.08	0.282
State-Trait Anxiety Inventory (STAI)-State	38.6 ±1.6	37.3 ±1.3	0.64	0.523
State-Trait Anxiety Inventory (STAI)-Trait	39.1 ±1.1	40.9 ±1.2	-1.17	0.245
Adult Autism Spectrum Quotient (ASQ)	19.7 ±0.8	21.1 ±1.0	-1.09	0.278
Social Provision Scale (SPS)	49.2 ±1.2	50.1 ±1.2	-0.55	0.587
Interpersonal Sensitivity Measure (IPSM)	94.0 ±1.2	96.9 ±1.4	-1.60	0.113
Revised Cheek and Buss Shyness Scale (RCBSS)	34.8 ±1.3	36.1 ±1.4	-0.72	0.476
Positive and Negative Affect Schedule (PANAS)-First time				
Positive	31.5 ±0.9	32.8 ±0.8	-1.10	0.277
Negative	19.4 ±0.7	21.9 ±1.0	-1.97	0.053
Positive and Negative Affect Schedule (PANAS)-Second time				
Positive	27.9 ±1.2	26.5 ±1.3	-0.82	0.414
Negative	10.5 ±0.4	11.2 ±0.7	-0.92	0.360
Individualism and Collectivism Scale (ICS)-HI	2.8 ±0.1	2.6 ±0.1	1.02	0.314
Individualism and Collectivism Scale (ICS)-VI	3.4 ±0.1	3.3 ±0.1	0.50	0.622
Individualism and Collectivism Scale (ICS)-HC	2.5 ±0.1	2.5 ±0.1	0.28	0.778
Individualism and Collectivism Scale (ICS)-VC	3.1 ±0.1	3.1 ±0.1	0.36	0.722
Fundamental Needs Questionnaire (FNQ)				
Belonging	3.89 ±0.2	3.81 ±0.2	0.27	0.788
Control	3.91 ±0.1	3.68 ±0.2	0.75	0.455
Self-esteem	4.94 ±0.2	4.95 ±0.2	-0.06	0.955
Meaningful-existence	3.99 ±0.2	3.69 ±0.2	1.19	0.237
Mood-bad	2.80 ±0.4	2.30 ±0.4	0.95	0.344
Mood-good	6.60 ±0.4	6.97 ±0.3	-0.81	0.421
Mood-sad	2.50 ±0.4	2.19 ±0.3	0.62	0.538
Mood-happy	6.68 ±0.3	7.03 ±0.3	-0.84	0.406
Mood-nervous	2.93 ±0.4	2.51 ±0.3	0.79	0.430
Mood-relax	7.00 ±0.4	7.43 ±0.4	-0.85	0.398
Mood-exciting	5.18 ±0.4	5.78 ±0.4	-1.14	0.258
Mood-drowsy	3.68 ±0.4	3.78 ±0.5	-0.19	0.853
Ancillary-enjoy	6.20 ±0.3	6.49 ±0.3	-0.62	0.540
Ancillary-angry	2.70 ±0.4	2.30 ±0.4	0.78	0.435
Involved	7.38 ±0.3	7.84 ±0.2	-1.26	0.211
Received ball	4.30 ±0.2	4.00 ±0.2	0.95	0.346
